# Comparison of nomogram for Primary Nonfunctional Pancreatic Neuroendocrine Tumors based on the 7th vs 8th edition of the AJCC cancer staging manual

**DOI:** 10.1371/journal.pone.0284930

**Published:** 2023-04-24

**Authors:** Xin Heng, Baijun Chen, Kui Zhao, Jun Li, Weiyu Wu, Yan Peng, Rui Zhong

**Affiliations:** 1 Department of Respiratory and Critical Care Medicine, Chengdu Second People’s Hospital, Chengdu, China; 2 Department of Gastroenterology, Clinical Medical College and The First Affiliated Hospital of Chengdu Medical College, Chengdu, China; 3 Department of Gastroenterology, The Affiliated Hospital of Southwest Medical University, Luzhou, China; Campus Bio Medico University, ITALY

## Abstract

**Background:**

Our study aimed to construct and validate prognostic nomograms for predicting survival for patients with Nonfunctional Pancreatic neuroendocrine tumor (NF-pNET).

**Methods:**

This retrospective study included 1824 patients diagnosed with NF-pNET in the Surveillance, Epidemiology and End Results database between 2004 and 2016. Randomization divided the patients into training (n = 1278) and validation (n = 546) cohorts. Prognostic factors were determined using Cox regression analyses, nomograms based on AJCC 7th and 8th staging system were constructed separately. The prediction models were validated using internal validation and external validation.

**Results:**

Age, year of diagnosis, primary tumor site, grade, 7th or 8th TNM stage, surgery, tumor size were determined as prognostic indicator to construct two nomograms. Harrell’s concordance index (C-index) of two nomograms exhibited a clinical predictive ability of 0.828 (95%CI, 0.808~0.849) vs 0.828 (95% CI, 0.808~0.849) in the internal verification. The c-index in the external validation was 0.812 (95%CI, 0.778~0.864) vs 0.814 (95% CI, 0.779~0.848). The predictive power of the two nomograms is comparable.

**Conclusions:**

Our nomogram may be a effective tool for predicting overall survival in patients with NF-pNET. The AJCC 8th-edition system provides discrimination similar to that of the 7th-edition system.

## Introduction

Pancreatic neuroendocrine tumor (pNET) is rare tumor that arise from pancreatic neuroendocrine cells [1, 2]. pNET account for 1% to 2% of all pancreatic malignancies, and the incidence has been steadily increasing over the past few decades [3]. Although this type of malignancy varies widely in biological behavior, most pNET have typically low-malignant behavior and slow growth rates, with a few exhibiting high invasive capabilities [4]. Distant metastasis occurs in 60% to 80% of patients and it is also one of the strongest predictors of survival [5]. The 5-year survival rate of pNET patients with liver metastasis is 13%-54%, which is significantly lower than that of patients without liver metastasis (75%-99%) [6]. pNET and pancreatic cancer (PC) differ markedly in cellular origin, biological characteristics, and prognosis. The prognosis of pNET is significantly better than that of PC.

NF-pNET are the most prevalent pNET, and tumor resection can significantly improve long-term survival, with 5-year survival rates of 65–86% and 10-year survival rates of 45%-68% for patients with NF-pNET after surgical resection [7]. The European Neuroendocrine Tumor Society (ENETS) recommends chemoradiotherapy for NF-pNET with distant metastases of any grade [8]. The efficacy of chemoradiotherapy, however, was not well established. Despite the advent of targeted therapy for NF-pNET, chemoradiotherapy continues to have a controversial role.

A patient’s prognosis may also be affected by factors other than surgical treatment for NF-pNET, such as the degree of pathological differentiation, tumor size [9], marital status [10], chemo-radiotherapy, and other treatment methods. Nomograms are widely used for NF-pNET prediction at the moment, however, the variables included and the predictive value are different. The American Joint Committee on Cancer (AJCC) released the eighth edition of the staging manual for pancreatic neuroendocrine tumors in 2017, which has some changes compared with the seventh edition. The eighth edition staging is more simplified than the seventh edition, however no studies have confirmed the prognostic decision-making ability of the eighth edition staging for NF-pNET patients. In addition, there is no nomogram to combine other variables using the 8^th^ TNM stage. Consequently, our aim was to construct an accurate prognostic evaluation system for NF-pNET patients.

## Methods

### Data collection

Data on 1824 patients with NF-pNET were extracted from the SEER database using version 8.3.8 of the SEER*Stat software (https://seer.cancer.gov, Client-server: ssp://seerstat.imsweb.com:2039, and the incidence SEER 18 Regs Custom Data (with additional treatment fields), Nov 2018 Sub (1975–2016 varying)) were selected for analysis [11]. We queried the SEER program database for records from 2004 to 2016 using inclusion criteria of the follows codes: The primary tumor site was coded as pancreas (C25.0-C25.9), the coding of NF-pNET pathological tissue coded was Large cell neuroendocrine carcinoma (8013), Islet cell adenoma(8150), Neuroendocrine carcinoma (8246). All functional, atypical, and mixed tumors were excluded, as well as those designated carcinoid or enterochromaffin-like tumors. The following information were collected for each patient: Marital, race, sex, age at diagnosis, survival time, overall survival (OS), regional nodes positive and examined, lymph node dissection, surgery of the primary site, radiation therapy and chemotherapy, whether there was bone/brain/lung/liver metastasis, tumor size, extension, year of diagnosis, primary tumor site, grade, seven edition of the AJCC staging system. According to the definition in SEER official manual, survival time means the time from diagnosis and death or the last follow-up. OS was defined the time from the date of diagnosis to the death of any cause. Surgery of the primary site include local excision of tumor, partial pancreatectomy, total pancreatectomy, local or partial pancreatectomy and duodenectomy, total pancreatectomy and subtotal gastrectomy or duodenectomy, extended pancreatoduodenectomy, pancreatectomy. The following codes was used to convert the seventh edition of the AJCC to the eighth edition: bone/brain/lung/liver metastasis, Mets at DX-Distant LN (2016+), Mets at DX-Other (2016+), collaborative stage (CS) tumor size 2004–2015, CS extension 2004–2015, CS lymph nodes 2004–2015, CS metastases at DX 2004–2015, Lymph-vascular Invasion (2004+ varying by schema), CS Reg Node Eval (2004–2015), CS Mets Eval (2004–2015), Regional nodes positive and derived AJCC stage group (7th edition; 2010+). We set the exclusion criteria as follows: (1) patients with unknown or incomplete clinical data; (2) patients with undiagnosed tumor pathology; (3) patients under the age of 18; (4) patients with a survival time of less than 1 month (5) Patients with multiple primary cancers. Neither the SEER database nor the data used for analysis contain any identifying information about people or demographics. Therefore, the participation in this study did not require ethics approval or informed consent.

### Statistical analysis

R software version 4.1.3 was used for statistical operations (https://www.r-project.org). Cox proportional hazards regression model used univariate and multivariate analysis to find its independent prognostic risk, P<0.05 was considered statistically significant. The best cutoff value of tumor size was determined by time-dependent receiver operating characteristic (ROC) curve. R software was used to construct a nomogram based on these independent risk factors. OS was measured at 1-, 3-, and 5-years according to the nomogram. In internal validation and external validation, harrell’s concordance indexes (C-indexes) and calibration curves were used to verify the predicted effect of the nomogram. The nomogram of 7^th^ TNM staging systems and 8^th^ TNM staging systems were compared using decision curves analysis (DCA) [12] and time-dependent ROC [13] curve. The “compare” function was used to compare the difference in the area under the ROC curve. The R software packages we used in the analysis include caret, foreign, survival, rms, pROC, Hmisc and rmda, ggDCA, timeROC, etc.

## Results

### Characteristics in training and validation cohorts

The R software caret package was used to divide the 1824 NF-pNET patients into the training cohort (1278 cases) and the validation cohort (546 cases) according to the ratio of 7:3. The demographic and clinicopathologic characteristics of patients in the training and validation cohorts were listed in Table 1. In the training set, the mean age of the patients was 59.1 years, the main population was white ethnicity (78.2%), the majority of patients were diagnosed with NF-pNET after 2011 (56.5%), the primary site of the tumor was mainly in the head (33.0%) and tail (33.3%) of the pancreas. The grade of tumor differentiation includes well differentiated (Grade1), moderately differentiated (Grade2), poorly differentiated (Grade3), well differentiated tumors (Grade1) make up the majority (65.3%). The seventh edition of the AJCC staging includes IA, IB, IIA, IIB, III, IV, and the eighth edition includes I, II, III, IV. Median survival of patients in the training Cohorts was 48 months. The characteristics of the patients in the training and external validation cohorts were similar, suggesting a good outcome for the cohort split.

**Table 1:** The demographic and clinicopathological characteristics of the training and validation cohorts.

Variable	training Cohorts	validation Cohorts	P-value
	n = 1278	n = 546	
Age	59.1±13.5	57.8±14.4	0.068
Race			0.805
White	1000 (78.2%)	429 (78.6%)	
Black	150 (11.7%)	59 (10.8%)	
Other	128 (10.0%)	58 (10.6%)	
Sex			0.793
Male	682 (53.4%)	287 (52.6%)	
Female	596 (46.6%)	259 (47.4%)	
Year of diagnosis			0.571
2004~2010	556 (43.5%)	229 (41.9%)	
2011~2016	722 (56.5%)	317 (58.1%)	
Primary site			0.308
Head of pancreas	422 (33.0%)	182 (33.3%)	
Body of pancreas	163 (12.8%)	85 (15.6%)	
Tail of pancreas	425 (33.3%)	179 (32.8%)	
Other	268 (21.0%)	100 (18.3%)	
Grade			0.568
G1	834 (65.3%)	363 (66.5%)	
G2	256 (20.0%)	98 (17.9%)	
G3	188 (14.7%)	85 (15.6%)	
AJCC Stage Group, 7th			0.261
IA	219 (17.1%)	112 (20.5%)	
IB	281 (22.0%)	105 (19.2%)	
IIA	126 (9.9%)	46 (8.4%)	
IIB	245 (19.2%)	102 (18.7%)	
III	48 (3.8%)	15 (2.7%)	
IV	359 (28.1%)	166 (30.4%)	
AJCC Stage Group, 8th			0.162
I	212 (16.6%)	108 (19.8%)	
II	372 (29.1%)	141 (25.8%)	
III	335 (26.2%)	131 (24.0%)	
IV	359 (28.1%)	166 (30.4%)	
Primary site surgery			0.805
No	294 (23.0%)	122 (22.3%)	
Yes	984 (77.0%)	424 (77.7%)	
Lymph node dissection			0.917
No	451 (35.3%)	188 (34.4%)	
1~3	148 (11.6%)	66 (12.1%)	
≥4	679 (53.1%)	292 (53.5%)	
Radiation therapy			1.000
No	1200 (93.9%)	513 (94.0%)	
Yes	78 (6.1%)	33 (6.0%)	
Chemotherapy			0.426
No	1012 (79.2%)	442 (81.0%)	
Yes	266 (20.8%)	104 (19.0%)	
Tumor size, mm	35 (22~56)	35 (20~55)	0.533
Marital status			0.255
Unmarried	367 (28.7%)	172 (31.5%)	
Married	911 (71.3%)	374 (68.5%)	
Survival time, month	48 (23~74)	46.5 (26~73)	0.861
Overall survival (OS)	865 (67.7%)	364 (66.7%)	0.711

### Screening for predictive factors

The univariate Cox regression revealed that several factors might influence the OS of patients with NF-pNET (P<0.05). The identified factors were then subjected to multivariate Cox regression analyses to establish independent prognostic variables (P<0.05). Univariate Cox regression analysis shows that the following factors have an impact on prognosis: age, race, primary site, grade, AJCC 7th Stage Group, AJCC 8th Stage Group, surgery, lymph node dissection, chemotherapy, radiotherapy, tumor size. After multivariate Cox regression analysis, age, primary tumor site, grade, AJCC 7th Stage Group, surgery were independent prognostic factors for OS. Surgical treatment, TNM stage, and grade showed an independent factor that significantly affected the prognosis for OS (Table 2). Then, the Cox model showed the tumor size also an independent prognostic factor based on the corrected AJCC 8th staging system (Table 3).

**Table 2:** Univariate and multivariate Cox regression analysis for OS in NF-pNET patients based on AJCC 7th in training cohorts.

Variable	Univariate analysis	P-value	Multivariate analysis	P-value
	HR(95%CI)		HR(95%CI)	
Age	1.03 (1.02–1.04)	<0.001	1.02 (1.01–1.03)	<0.001
Race				
White	reference			
Black	1.43 (1.09–1.89)	0.011	1.12 (0.84–1.48)	0.435
Other	0.99 (0.71–1.39)	0.791	1.14 (0.81–1.61)	0.440
Sex				
Male	reference			
Female	0.83 (0.69–1.01)	0.066		
Year of diagnosis				
2004~2010	reference			
2011~2016	0.87 (0.71–1.07)	0.194		
Primary site				
Head of pancreas	reference		reference	
Body of pancreas	0.65 (0.47–0.90)	0.010	0.83 (0.59–1.15)	0.261
Tail of pancreas	0.54 (0.42–0.69)	<0.001	0.67 (0.52–0.87)	0.002
Other	0.82 (0.64–1.05)	0.113	0.82 (0.63–1.07)	0.142
Grade				
G1	reference		reference	
G2	1.55 (1.19–2.01)	0.001	1.21 (0.93–1.59)	0.154
G3	7.96 (6.38–9.93)	<0.001	4.18 (3.24–5.41)	<0.001
AJCC Stage Group,7th				
IA	reference		reference	
IB	1.79 (1.02–3.13)	0.043	1.32 (0.73–2.38)	0.360
IIA	4.03 (2.29–7.11)	<0.001	2.64 (1.43–4.89)	0.002
IIB	2.89 (1.69–4.96)	<0.001	1.99 (1.10–3.61)	0.024
III	7.67 (4.12–14.28)	<0.001	2.88 (1.44–5.75)	0.003
IV	11.71 (7.15–19.18)	<0.001	4.32 (2.45–7.65)	<0.001
Primary site surgery				
No	reference		reference	
Yes	0.15 (0.12–0.18)	<0.001	0.34 (0.22–0.52)	<0.001
Lymph node dissection				
No	reference		reference	
1~3	0.31 (0.22–0.45)	<0.001	1.09 (0.66–1.80)	0.738
≥4	0.28 (0.23–0.35)	<0.001	0.85 (0.56–1.29)	0.449
Radiation therapy				
No	reference		reference	
Yes	2.55 (1.88–3.45)	<0.001	1.06 (0.77–1.46)	0.724
Chemotherapy				
No	reference		reference	
Yes	3.69 (3.03–4.50)	<0.001	0.85 (0.67–1.09)	0.200
Tumor size, mm				
<30	reference		reference	
≥30	2.72 (2.14–3.46)	<0.001	1.27 (0.97–1.66)	0.079
Marital status				
Unmarried	reference			
Married	0.86 (0.70–1.06)	0.164		

**Table 3:** Univariate and multivariate Cox regression analysis for OS in NF-pNET patients based on AJCC 8th in training cohorts.

Variable	Univariate analysis	P-value	Multivariate analysis	P-value
	HR(95%CI)		HR(95%CI)	
Age	1.03 (1.02–1.04)	<0.001	1.02 (1.01–1.03)	<0.001
Race				
White	reference			
Black	1.43 (1.09–1.89)	0.011	1.13 (0.86–1.50)	0.379
Other	0.99 (0.71–1.39)	0.971	1.14 (0.81–1.60)	0.455
Sex				
Male	reference			
Female	0.83 (0.69–1.01)	0.066		
Year of diagnosis				
2004~2010	reference			
2011~2016	0.87 (0.71–1.07)	0.194		
Primary site				
Head of pancreas	reference		reference	
Body of pancreas	0.65 (0.47–0.90)	0.010	0.83 (0.59–1.16)	0.278
Tail of pancreas	0.54 (0.42–0.69)	<0.001	0.66 (0.51–0.86)	0.002
Other	0.82 (0.64–1.05)	0.133	0.85 (0.65–1.10)	0.210
Grade				
G1	reference		reference	
G2	1.55 (1.19–2.01)	0.001	1.21 (0.93–1.59)	0.154
G3	7.96 (6.38–9.93)	<0.001	4.23 (3.27–5.47)	<0.001
AJCC Stage group,8th				
I	reference		reference	
II	2.13 (1.25–3.63)	0.005	1.55 (0.88–2.74)	0.128
III	3.54 (2.12–5.93)	<0.001	2.10 (1.18–3.73)	0.011
IV	11.39 (6.96–18.66)	<0.001	4.02 (2.28–7.11)	<0.001
Primary site surgery				
No	reference		reference	
Yes	0.15 (0.12–0.18)	<0.001	0.33 (0.22–0.51)	<0.001
Lymph node dissection				
No	reference		reference	
1~3	0.31 (0.22–0.45)	<0.001	1.11 (0.67–1.84)	0.674
≥4	0.28 (0.23–0.35)	<0.001	0.86 (0.56–1.30)	0.465
Radiation therapy				
No	reference		reference	
Yes	2.55 (1.88–3.45)	<0.001	1.07 (0.78–1.48)	0.662
Chemotherapy				
No	reference		reference	
Yes	3.69 (3.03–4.50)	<0.001	0.88 (0.69–1.13)	0.314
Tumor size, mm				
<30	reference		reference	
≥30	2.72 (2.14–3.46)	<0.001	1.32 (1.01–1.72)	0.044
Marital status				
Unmarried	reference			
Married	0.86 (0.70–1.06)	0.164		

### Risk prediction nomogram development and validation

**Fig 1:**
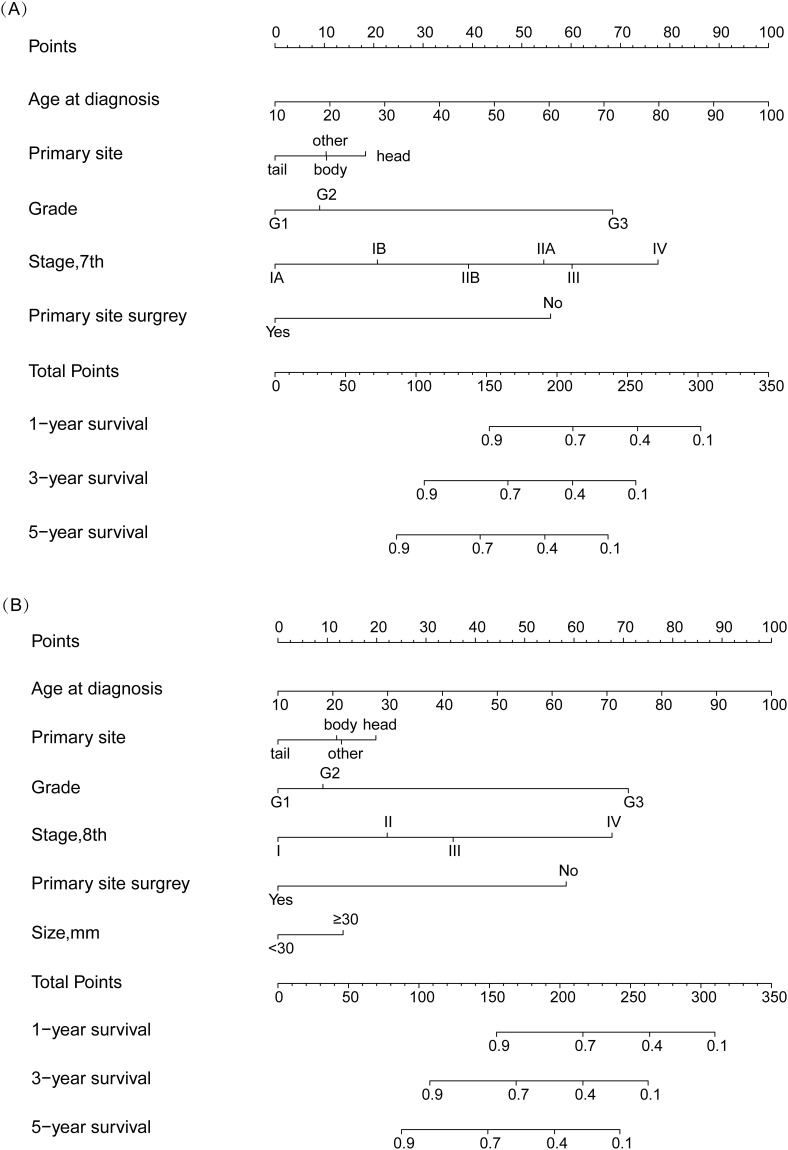
Nomograms for predicting the 1,3- and 5-year OS of NF-pNET patients (A: NomogramA; B: NomogramB).

**Fig 2:**
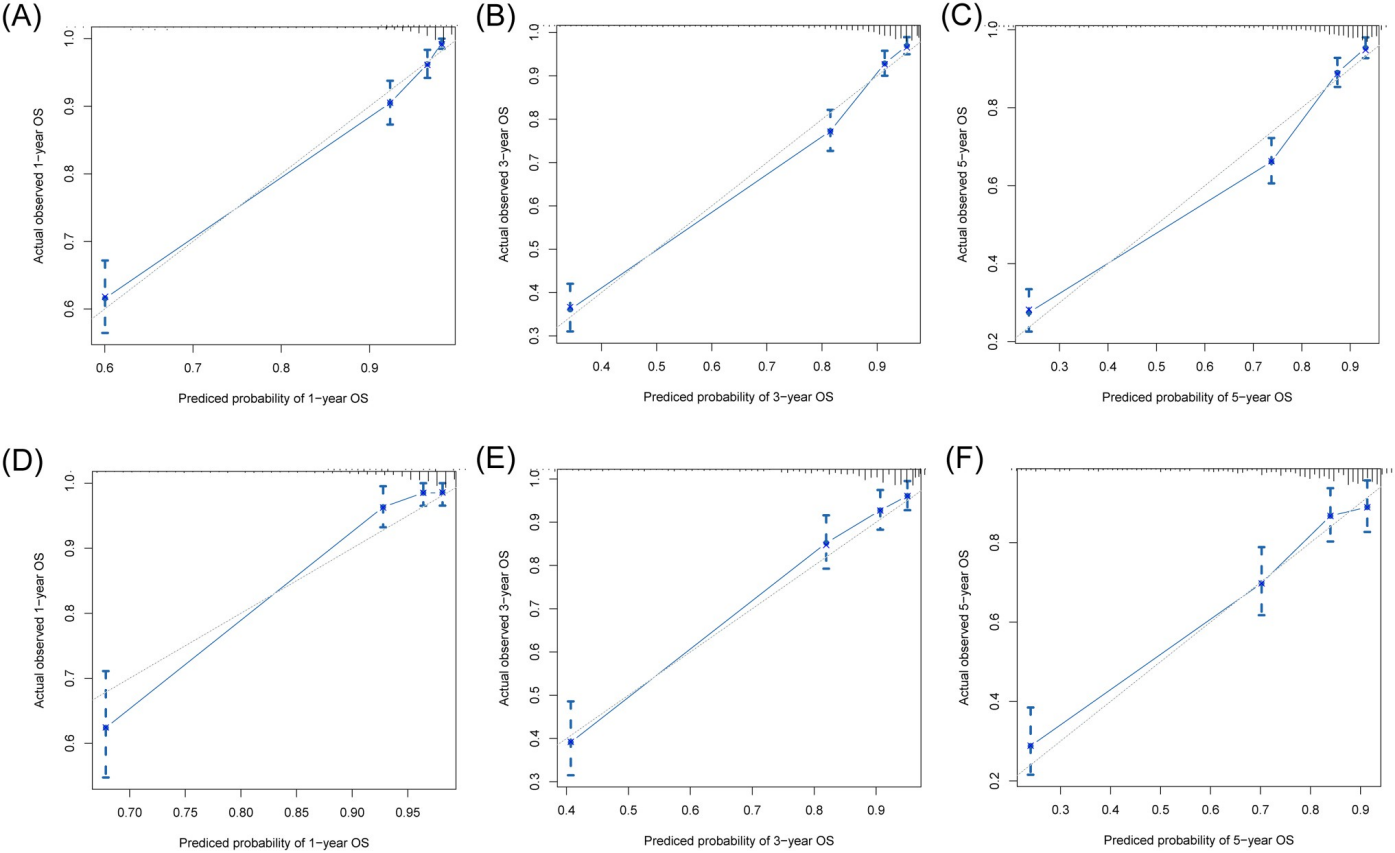
Calibration curves of the NomogramA using the training cohort for predicting the 1- (A), 3- (B), and 5- (C) year OS rates of patients with NF-pNET. Calibration curves of the NomogramA using the validation cohort for predicting the 1- (D), 3- (E), and 5- (F) year OS rates of patients with NF-pNET.

**Fig 3:**
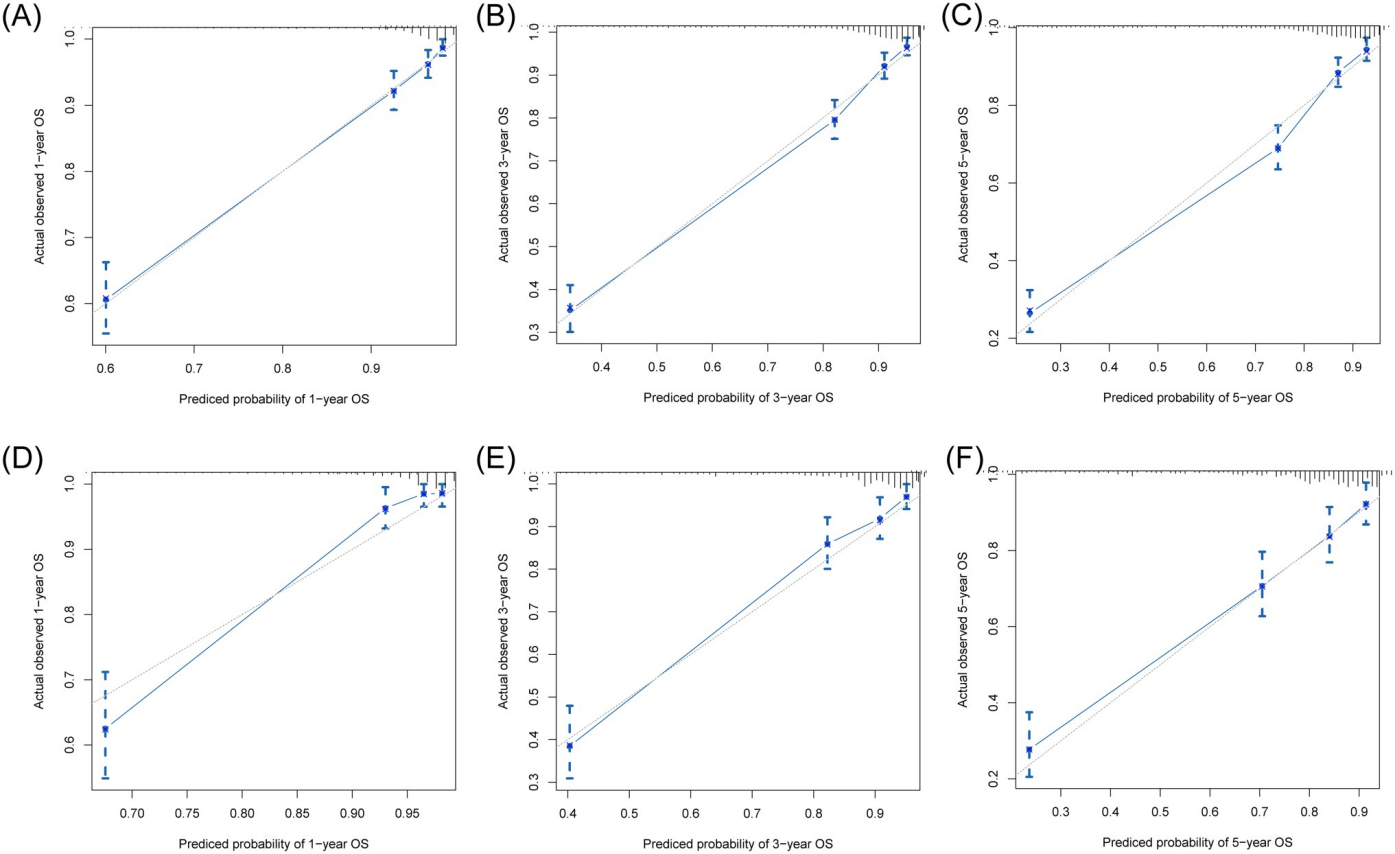
Calibration curves of the NomogramB using the training cohort for predicting the 1- (A), 3- (B), and 5- (C) year OS rates of patients with NF-pNET. Calibration curves of the NomogramB using the validation cohort for predicting the 1- (D), 3- (E), and 5- (F) year OS rates of patients with NF-pNET.

We constructed two nomograms based on the 7th and 8th AJCC staging system, respectively (named NomogramA and NomogramB, respectively). The NomogramA comprised 5 prognostic factors: Age, primary tumor site, grade, 7^th^ TNM stage, surgery. The NomogramB comprised 6 prognostic factors: Age, primary tumor site, grade, 8^th^ TNM stage, surgery, tumor size. Each prognostic factor will have a corresponding weighted score, and the sum of these factors were the total score for the patient. For each NF-pNET patient, higher total points indicated a lower of 1,3- and 5-year survival rates (Fig 1). First, internal and external validation of NomogramA was performed, the C-index calculated by the bootstrap self-sampling method was 0.828 (95%CI, 0.808~0.849) in internal validation, the C-index was 0.812 (95%CI, 0.778~0.846) in external validation. Then, internal and external validation of NomogramB was performed, the C-index was 0.828 (95% CI, 0.808~0.849) in internal validation and 0.814 (95% CI, 0.779~0.848) in external validation. This indicates good predictability of both nomograms. In addition, the calibration curve was similar to the standard curve in predicting the 1, 3- and 5-year survival rates of patients, indicating good predictive ability of the nomogram (Figs 2 and 3).

### Decision curve analysis of nomogram

**Fig 4:**
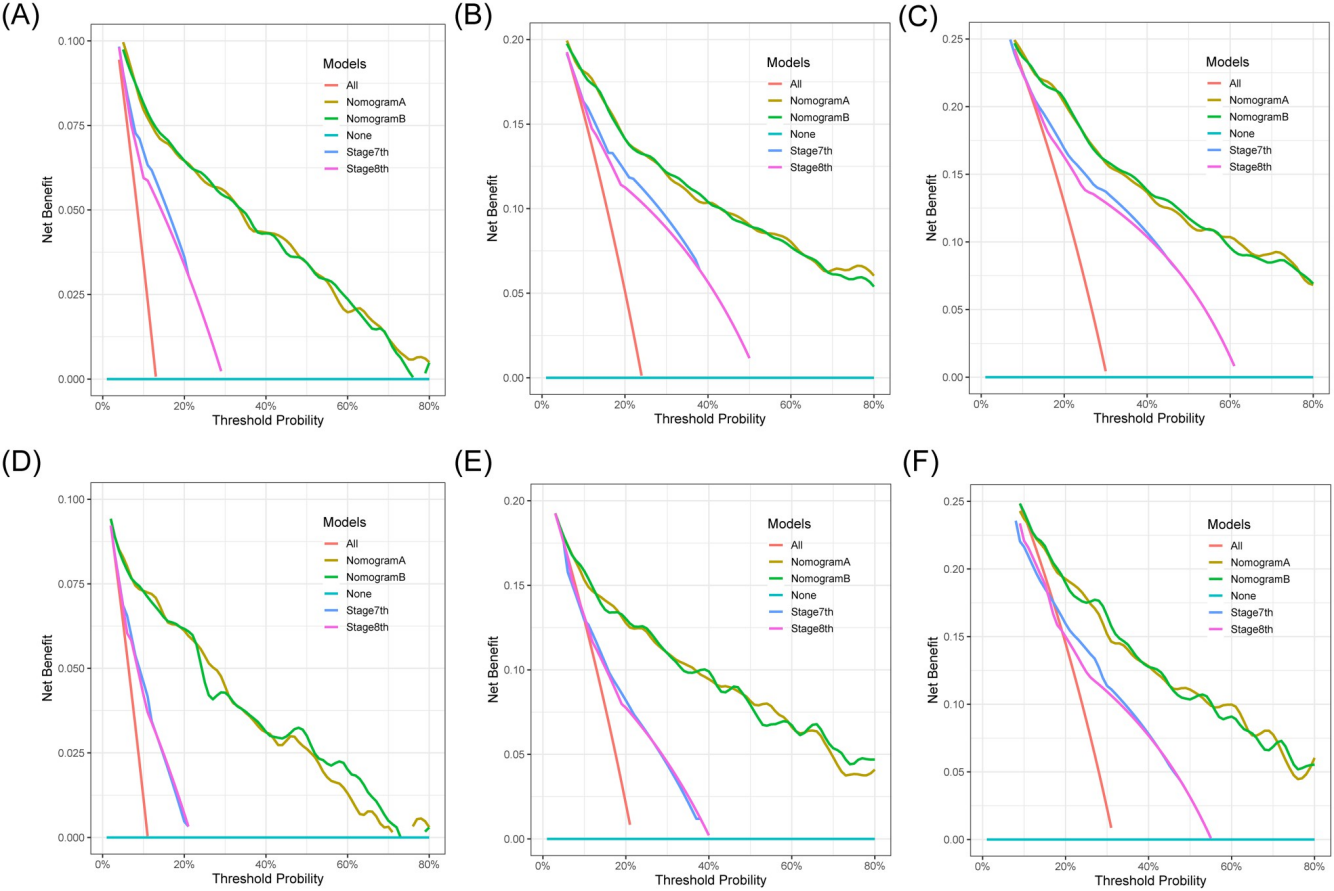
Decision curve analysis for the 1- (A), 3- (B), and 5- (C) year OS rates of patients with NF-pNET in training cohort. Decision curve analysis for the 1- (D), 3- (E), and 5- (F) year OS rates of patients with NF-pNET in validation cohort.

Decision Curve Analysis is an assessment method that evaluates the degree of patient benefit. The analysis was done with the R software rmda package. Four DCA curves with different colors represent four different clinical diagnosis models. In addition, there were two reference lines. The horizontal red-line means no intervention in all samples, the net benefit is zero, and the slanted black-line means that all samples are subject to intervention. The results show that NomogramA and NomogramB have almost the similar clinical benefit rate, and both are significantly better than the 7th TNM staging system or 8th TNM staging system (Fig 4). Therefore, the revised AJCC 8th-edition system provides discrimination similar to that of the 7th-edition system.

### Time-dependent receiver operating characteristic curve analysis of nomogram

**Fig 5:**
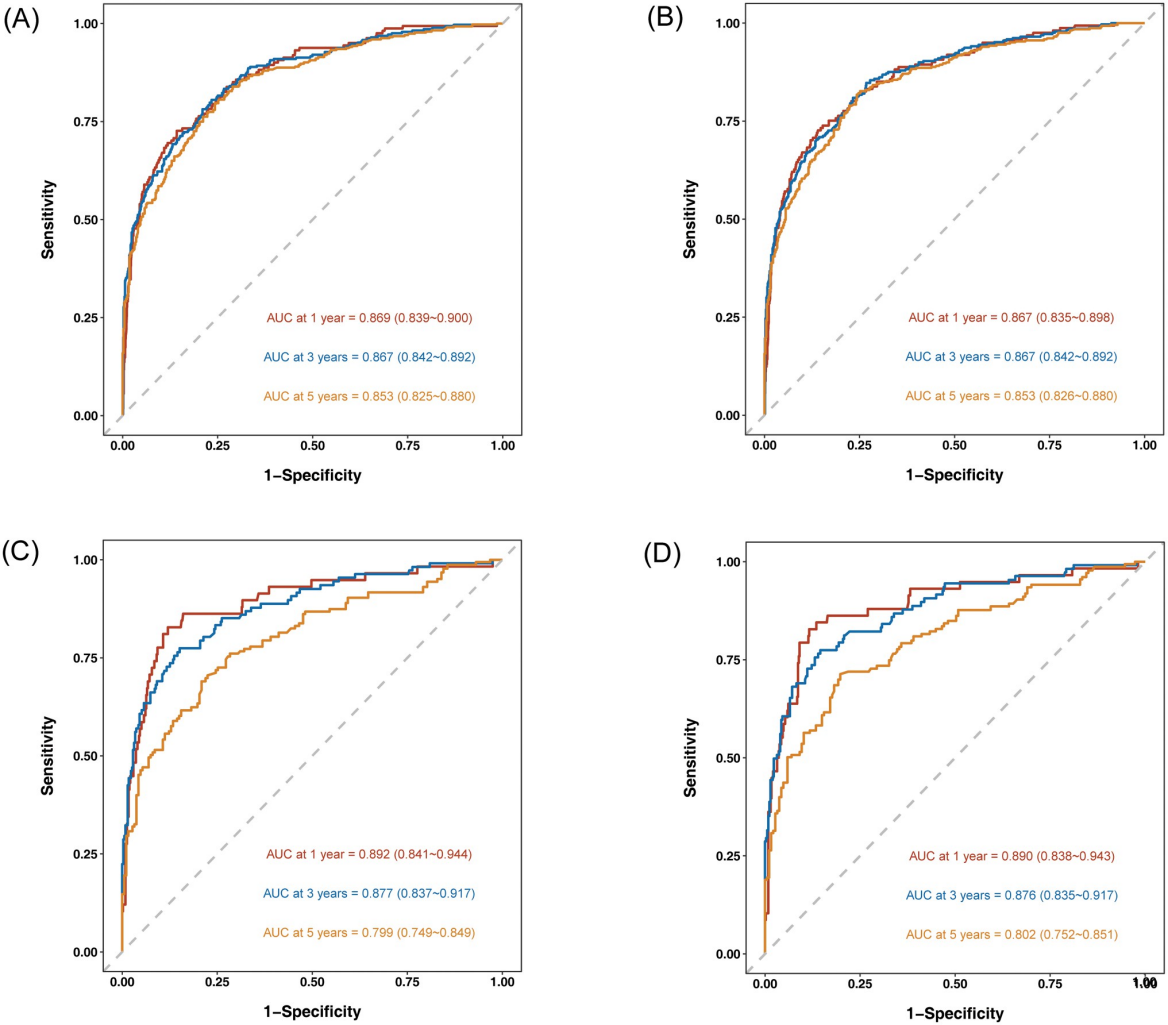
Time-dependent ROC curves for the 1-, 3-, and 5-year OS probability patients with NF-pNET in training cohort (A:NomogramA; B: NomogramB). Time-dependent ROC curves for the 1-, 3-, and 5-year OS probability patients with NF-pNET in validation cohort (C:NomogramA; D: NomogramB).

The time-dependent ROC curve was used to evaluate the prognostic performance for nomograms. The area under the curve (AUC) suggested great predictive power in terms of 1-, 3- and 5-year survival for the OS rates of patients with NF-pNET in training cohort and validation cohort. The results of the two cohorts were basically the same. In addition, the results also showed that the AUC of NomogramA and NomogramB are basically the same, indicating that the two models have comparable predictive capabilities (Fig 5). Finally, Compared the AUC of NomogramA and NomogramB through the R software “compare” function. The results showed that the difference between area of AUC was not statistically significant (all adjusted P-value > 0.05), and the AUC of the two models could be considered equal (Table 4). In other words, the results proved again that the revised AJCC 8th-edition system provides discrimination similar to that of the 7th-edition system.

**Table 4:** The difference between area of AUC for the 1-, 3-, and 5-year OS probability patients with NF-pNET in training cohort and validation cohort.

AUC*	training Cohorts		validation Cohorts	
	P(Non-adjusted)	P(Adjusted)	P(Non-adjusted)	P(Adjusted)
1year	0.606	0.872	0.472	0.836
3year	0.759	0.966	0.854	0.996
5year	0.428	0.685	0.657	0.954

* The difference between area of AUC for NomogramA and NomogramB.

## Discussion

In this study, we included NF-pNET patients from the SEER database, and univariate and multivariate Cox regression analysis revealed that age, primary tumor site (Head of pancreas), non-surgical resection, G3, tumor size ≥30mm and TNM stage were independently associated with poor prognosis in NF-pNET. Second, we found that the nomogram constructed based on Cox regression analysis had a strong predictive ability for the survival of patients with NF-pNET. And finally, our study confirmed that the updated AJCC 8th edition staging has the same predictive power for the prognosis of NF-pNET as AJCC 7th edition staging comparison.

pNET are a class of tumors with characteristic neuroendocrine differentiation and expression of neuroendocrine markers, with an annual incidence of 0.19/100,000 to 0.32/100,000 and approximately 2–5% of all pancreatic malignancies. The functional tumors represent approximately 10–30% of pNET, NF-pNET comprise the remaining 50–80% [4]. Early detection of patients with NF-pNET and appropriate clinical intervention can significantly improve patient outcomes [14]. Comprehensive treatment based on surgery is the best way to achieve good long-term prognosis for patients with NF-pNET. The formulation of surgical strategies should comprehensively consider the patient’s systemic status, tumor function and biological characteristics, and carefully evaluate the risks and benefits of surgery [15]. Theoretically, surgical resection could provide the greatest benefit compared to chemotherapy and radiotherapy. Our study produced similar results.

Mei concluded that in 3011 patients, pNET were more common in the pancreatic body and tail (62.94%) than in the pancreatic head (37.06%). Tumors in the head of the pancreas were larger, more likely to have positive lymph nodes, and were more prone to locally advanced and distant invasion. Pancreatic head pNET with a size of 21–40 mm have a worse prognosis than body/tail pNET. Pancreatic body/tail tumors have better overall survival for NF-pNET [16]. Our study also found that tumor primary site was an independent prognostic factor, and also found that tumor size was significant in univariate and multivariate regression analysis. These findings were consistent with previous studies.

There is no doubt that age is closely related to the prognosis of many diseases, and organ aging coupled with decreased immune function may lead to tumor recurrence. Elderly patients with NF-pNET may not tolerate surgery, radiotherapy, and chemotherapy, so they may be less compliant with anticancer therapy. In addition, factors such as other diseases and economic burden often need to be considered in such patients [17]. Therefore, age is associated with the prognosis of patients with NF-pNET, and our study reconfirmed the previous results.

Although most patients are insensitive to chemoradiotherapy, chemoradiotherapy remains an alternative treatment option for patients with advanced pNET [18], but our study showed that radiotherapy did not help improve OS. Some previous studies have shown that radiotherapy is beneficial to the prognosis of patients with pNET [19]. Peptide receptor radionuclide therapy (PRRT) for metastatic pNET can effectively delay tumor growth and can be used as a first-line therapy [20]. The University of Maryland School of Medicine concluded that local radiation therapy may convert initially unresectable locally-advanced tumors to disease amenable to surgical resection, which would theoretically improve local control [21]. Murase found surgery after sunitinib administration to improve survival of patients with advanced pancreatic neuroendocrine neoplasms [22]. Whether chemoradiotherapy can improve the prognosis of patients with distant metastases still needs to be confirmed by prospective studies [23]. The current increase in targeted therapy and immunosuppressive therapy provides new treatment avenues for patients who have lost the opportunity for surgery, but may only be effective in a select group of people. Previous studies have also found that combination therapy does not improve overall survival for pNET [24, 25].

Tumor grade is determined according to the degree of tumor tissue anaplastic, including the degree of differentiation, arrangement, number of mitoses and local infiltration of cancer cells. It can provide reference basis for clinical treatment and prognosis estimation. Generally divided into four or three grades, the higher the grade of the tumor, the higher the degree of malignancy. Less differentiated tumors have more aggressive biology, leading to earlier local and distant metastasis [26]. Tumor grade has been included as part of the eighth edition of the AJCC staging system for prostate cancer and sarcomas because of its ability to distinguish differences in overall survival among these patients [27]. Our analysis suggests that tumor grade was a good prognostic factor for survival in NF-pNET, and subsequent studies could be considered for inclusion in the AJCC staging system.

The eighth edition of the AJCC Staging Manual was released in October 2016, and officially applied globally in 2018. The new PNET staging system has significant revisions compared to the 7th edition, including an updated definition of the T classification and a reduction in the A/B classification of stages I/II in staging. Many studies have investigated the effectiveness of the 8th edition of the AJCC staging system for PC, but PNET has not been investigated. Our studies showed AJCC 8th-edition system provides discrimination similar to that of the 7th-edition system. Our results showed that the Stage IIA patients had a worse prognosis than stage IIB patients, which may indicate subdivision of stage I/ II into A and B subgroups in the 7th edition of AJCC does not better distinguish patients with different prognoses, but makes the clinical application of the staging system more cumbersome. The 8th-edition of the TNM staging system demonstrated a more equal distribution among stages and simpler on pNET compared with the 7th-edition.

This study has several limitations. First, our study was retrospective and the data came from public databases, So there may be some bias in the statistics. Second, the SEER database lacks some specific chemoradiotherapy information and does not provide additional information on tumor grade, which may have affected our results. Finally, patient information in the SEER database is from the United States, which may be underrepresented globally, and our research model lacks validation from external datasets. More well-designed prospective studies with large samples are needed to further support our view.

## Conclusion

The update AJCC 8th-edition system provides discrimination similar to that of the 7th-edition system. In addition, the proposed nomogram containing TNM stage, age, primary site of tumor, tumor size, surgery and grade reveals a superior prognostic model for NF-pNET patients.

## Supporting information

S1 File NF-PNET. (XLSX)
